# EGFR modulates monounsaturated fatty acid synthesis through phosphorylation of SCD1 in lung cancer

**DOI:** 10.1186/s12943-017-0704-x

**Published:** 2017-07-19

**Authors:** Jiqin Zhang, Fei Song, Xiaojing Zhao, Hua Jiang, Xiuqi Wu, Biao Wang, Min Zhou, Mi Tian, Bizhi Shi, Huamao Wang, Yuanhui Jia, Hai Wang, Xiaorong Pan, Zonghai Li

**Affiliations:** 1 State Key Laboratory of Oncogenes and Related Genes, Shanghai Cancer Institute, Renji Hospital, Shanghai Jiao Tong University School of Medicine, No.25/Ln2200, XieTu Road, Shanghai, 200032 People’s Republic of China; 2 Shanghai Key Laboratory of Regulatory Biology, the Institute of Biomedical Sciences and School of Life Sciences, East China Normal University, Shanghai, 200241 China; 3 Department of Thoracic Surgery, Renji Hospital, Shanghai Jiao Tong University School of Medicine, Shanghai, 200127 China; 4 Clinical and Translational Research Center, Shanghai First Maternity and Infant Hospital, Tongji University School of Medicine, Shanghai, 200040 China; 5 Lester and Sue Smith Breast Center, Baylor College of Medicine, One Baylor Plaza, Houston, TX 77030 USA; 6 Department of Molecular and Cellular Biology, Baylor College ofMedicine, One Baylor Plaza, Houston, TX 77030 USA

## Abstract

**Background:**

Epidermal growth factor receptor (EGFR), a well-known oncogenic driver, contributes to the initiation and progression of a wide range of cancer types. Aberrant lipid metabolism including highly produced monounsaturated fatty acids (MUFA) is recognized as a hallmark of cancer. However, how EGFR regulates MUFA synthesis in cancer remains elusive. This is the focus of our study.

**Methods:**

The interaction between EGFR and stearoyl-CoA desaturase-1 (SCD1) was detected byco-immunoprecipitation. SCD1 protein expression, stability and phosphorylation were tested by western blot. The synthesis of MUFA was determined by liquid chromatography-mass spectrometry. The growth of lung cancer was detected by CCK-8 assay, Annexin V/PI staining, colony formation assay and subcutaneous xenograft assay. The expression of activated EGFR, phosphorylated and total SCD1 was tested by immunohistochemistry in 90 non-small cell lung cancersamples. The clinical correlations were analyzed by Chi-square test, Kaplan-Meier survival curve analysis and Cox regression.

**Results:**

EGFR binds to and phosphorylates SCD1 at Y55. Phosphorylation of Y55 is required for maintaining SCD1 protein stability and thus increases MUFA level to facilitate lung cancer growth. Moreover, EGFR-stimulated cancer growth depends on SCD1 activity. Evaluation of non-small cell lung cancersamples reveals a positive correlation among EGFR activation, SCD1 Y55 phosphorylation and SCD1 protein expression. Furthermore, phospho-SCD1 Y55 can serve as an independent prognostic factor for poor patient survival.

**Conclusions:**

Ourstudy demonstrates that EGFR stabilizes SCD1 through Y55 phosphorylation, thereby up-regulating MUFA synthesis to promote lung cancer growth. Thus, we provide the first evidence that SCD1 can be subtly controlled by tyrosine phosphorylation and uncover a previously unknown direct linkage between oncogenic receptor tyrosine kinase and lipid metabolism in lung cancer. We also propose SCD1 Y55 phosphorylation as a potential diagnostic marker for lung cancer.

**Electronic supplementary material:**

The online version of this article (doi:10.1186/s12943-017-0704-x) contains supplementary material, which is available to authorized users.

## Background

An increasing number of studies suggest that altered lipid metabolism is one of new hallmarks of cancer in recent years [1, 2]. De novo synthesis of lipid, as the main composition of cell membrane, is abnormally fast in cancer cells to provide enough building blocks for rapid cell replication and growth [3–6]. Saturated fatty acids (SFA) and monounsaturated fatty acids (MUFA) are two major products during this process. Stearoyl-CoA desaturase-1 (SCD1) is a rate-limiting enzyme responsible for MUFA synthesis, which introduces a double bond in the cis-delta-9 position of a few saturated fatty acylCoAs [7]. SCD1 has been proven to be involved in sustaining rapid cell proliferation, evading cell apoptosis, facilitating cancer cell initiation and malignant transformation in various types of cancer [8–10]. It is noteworthy that the influence by SCD1 is closely associated with the change of MUFA level, because exogenous addition of MUFA is able to rescue the defects due to SCD1 abrogation under some conditions [11]. In line with the significance of SCD1 in cancer, highly expressed SCD1 has been found in diverse cancer types including lung, breast and prostate cancers when compared with normal tissues [12–17]. Furthermore, recent studies disclose that high level of SCD1 protein expression is correlated with poor patient prognosis in breast cancer and hepatocellular carcinoma [18, 19].

The current understanding of SCD1 regulation is mainly focus on gene transcription. There are a number of transcription factor binding sites in the region of SCD1 promoter. It has been reported that sterol response element-binding protein (SREBP), peroxisome proliferator-activated receptor (PPAR), LXR, NF-1 and AP-2 modulate the gene transcription of SCD1 [20–22]. On the other hand, one study indicates that the protein stability of SCD1 is regulated by ubiquitin proteasome dependent degradation [23]. However, how SCD1 is affected by other post-translational mechanisms has been poorly studied up to now.

As a typical cell membrane receptor, epidermal growth factor receptor (EGFR) is highly expressed in various types of cancer and identified as an oncogenic driver as well as a validated target for cancer therapy [24–29]. In recent years, increasing data indicate that EGFR plays direct roles in DNA replication, DNA repair, microRNA maturation and autophagy through phosphorylating critical factors [30–35]. Intriguingly, EGFR is also proved to regulate cancer metabolism by the finding that it keeps the intracellular level of glucose through maintaining the protein stability of sodium/glucose cotransporter 1 (SGLT1) in a kinase activity independent manner [36]. Nevertheless, whether EGFR directly affects lipid metabolism pathways still remains elusive.

In this study, we find that EGFR stabilizes SCD1 through Y55 phosphorylationto increase intracellular MUFA level and consequently promotes lung cancer growth. Furthermore, we reveal a clinical association among phospho-EGFR Y1092, phospho-SCD1 Y55, SCD1 protein expression and short patient survival in non-small cell lung cancer (NSCLC). Taken together, our findings uncover a novel mechanism that EGFR directly modulates MUFA synthesis to promote lung cancer growth.

## Results

### EGFR interacts with SCD1

**Fig. 1:**
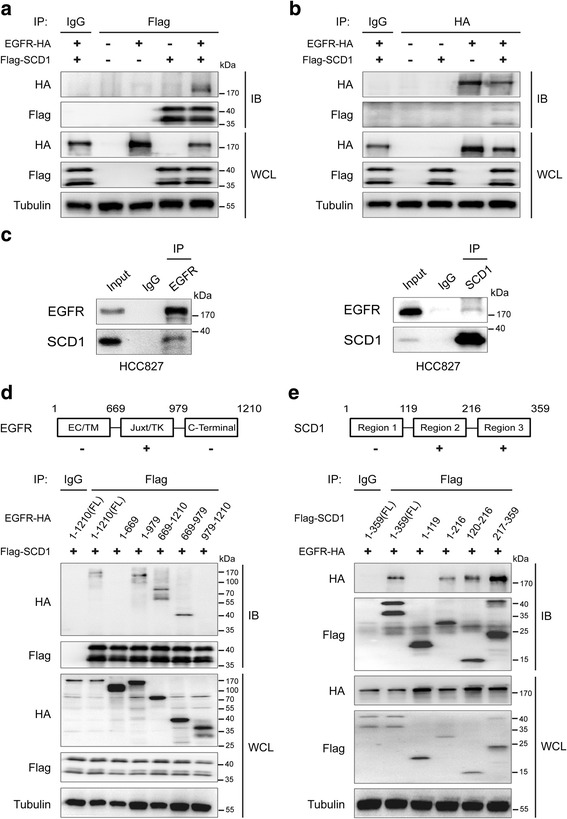
EGFR interacts with SCD1. **a** and **b** Lysates from 293 T cells exogenously expressing EGFR-HA (at C-terminus) and Flag-SCD1 (at N-terminus) were subjected to immunoprecipitation (IP) and immnuoblotting (IB) with the indicated antibodies. WCL, whole cell lysates. **c** Reciprocal immunoprecipitation and western blot analysis in HCC827 cells. Ten percentage of the lysate for immunoprecipitation is shown as input. **d** and **e** Lysates from 293 T cells exogenously co-expressing Flag-SCD1 and full-length or different functional domains of EGFR-HA (**d**), or co-expressing EGFR-HA and full-length or different regions of Flag-SCD1 (**e**) were subjected to IP/IB with the indicated antibodies. FL, full-length

Given that EGFR can act as anoncogenic driver and phosphorylate a variety of cancer-promoting factors, we speculated whether it is also capable of directly regulating the key enzymes involved in lipid metabolism. To test this hypothesis, we first determined the interaction between EGFR and SCD1, a rate-limiting enzyme responsible for MUFA synthesis. Co-immunoprecipitation assay in 293 T cells showed that there was a protein-protein interaction between EGFR and SCD1 (Fig. 1a and b). The lower band in gel, detectable by anti-Flag antibody, may represent a cleaved product of SCD1 which was reported previously [37, 38]. Their binding was also observed by using SCD1 with C-terminal Flag tag (data not shown).

It has been reported that EGFR and SCD1 play significant roles in lung cancer. Thus, we carried out reciprocal immunoprecipitation and western blot in several lung cancer cell lines (A549, HCC827 and H1975) to further validate the interaction. The results indicated that both wild-type and mutated EGFR (△746–750 in HCC827 cells, L858R and T790 M in H1975 cells) bound to SCD1 (Fig. 1c and Additional file 1: Figure S1A). To further examine whether other EGFR mutants also interact with SCD1, we made a series of EGFR constructs with mutations as ∆exon2–7 (EGFRvIII), ∆746–750, L858R and T790 M for immunoprecipitation/western blot analysis. Like wild type, all EGFR mutants were able to bind to SCD1 (Additional file 1: Figure S1B). To further clarify the regions of EGFR and SCD1 necessary for their interaction, we constructed several truncated mutants. The results of immunoprecipitation in 293 T cells showed that the juxtamembrane and tyrosine kinase domains of EGFR and two fragments of SCD1 (aa 120–216 and aa 217–359) were required for their binding (Fig. 1d and e). Altogether, these data suggest that EGFR can interact with SCD1.

### EGFR kinase activity maintains SCD1 protein stability and intracellular MUFA level in lung cancer

**Fig. 2:**
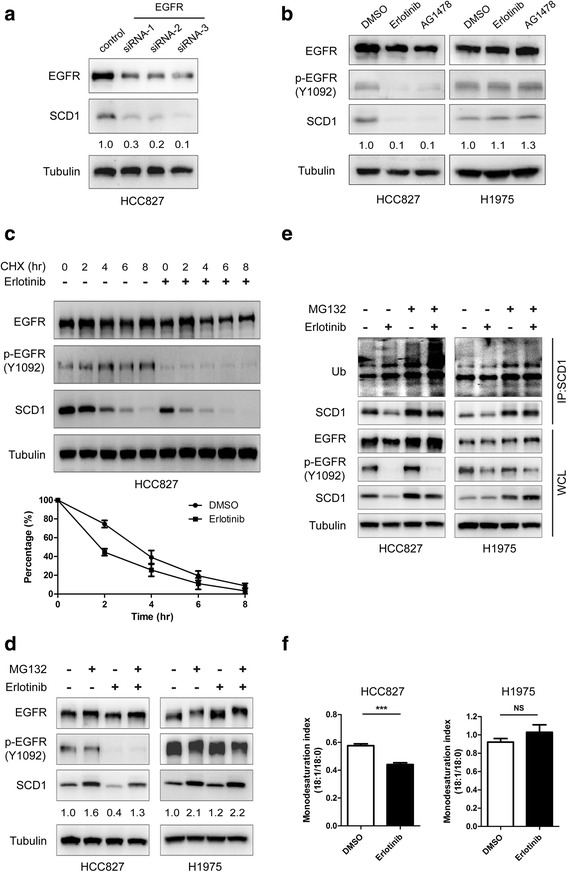
EGFR kinase activity is essential for maintenance of SCD1 protein stability and intracellular MUFA level in lung cancer. **a** HCC827 cells were transfected with different siRNAs against EGFR or a control scramble siRNA for 72 h, and the lysates were subjected to IB. **b** HCC827 and H1975 cells were treated with 0.1% dimethyl sulfoxide (DMSO) as control, erlotinib (1 μM) or AG1478 (1 μM) for 24 h and the lysates were blotted with the antibodies as indicated. **c** HCC827 cells were pre-incubated with 0.1% DMSO or erlotinib (1 μM) for 12 h and 100 μg/ml cycloheximide (CHX) was then added for the indicated time. The lysates were subjected to IB. Densitometry quantitative data (SCD1/Tubulin) are mean ± SEM from three independent experiments. **d** and **e** In the presence or absence of erlotinib (1 μM), HCC827 and H1975 cells were treated with MG132 (10 μM) for 12 h and the lysates were subjected to IB or IP/IB with the indicated antibody. **f** HCC827 and H1975 cells were incubated with 0.1% DMSO or erlotinib (1 μM) for 24 h. Total cell lipids were extracted and the ratio of monounsaturated fatty acids (18:1) to saturated fatty acid (18:0) was determined by liquid chromatography-mass spectrometry (LC-MS). Data are mean ± SD (*n* = 3). ***p < 0.001; NS, nonsignificance. Densitometry quantification of SCD1/Tubulin (**a**, **b** and **d**) level is shown

To investigate whether EGFR regulates SCD1, we first detected the alteration of SCD1 protein level after knockingdown EGFR by specific small interfering RNAs (siRNAs) or overexpressing EGFR. It was observed that the protein expressions of EGFR and SCD1 were positively correlated each other (Fig. 2a, Additional file 1: Figure S2A and B). To further understand if kinase activity is indispensable for EGFR to modulate SCD1 protein expression, we used erlotinib and AG1478 (EGFR tyrosine kinase inhibitors, TKIs) in HCC827 and H1975 cells, which are drug-sensitive and resistant, respectively. As shown in Fig. 2b, SCD1 protein level was significantly down-regulated along with the decrease of EGFR kinase activity in HCC827 cells whereas no obvious changes were observed in H1975 cells. Consistent results were also obtained in A549 cells upon EGF stimulation or TKIs treatment (Additional file 1: Figure S2C and D). These results imply that tyrosine kinase activity is important for EGFR to regulate SCD1 protein expression.

Next, we explored whether EGFR kinase activity modulates the protein stability of SCD1. In HCC827 cells, SCD1 protein level was compared in the presence or absence of erlotinib after the treatment of cycloheximide (CHX) which inhibited protein synthesis. As shown in Fig. 2c, the protein degradation of SCD1 was markedly faster when EGFR was inactivated. Additionally, we found that SCD1 protein level was rescued more significantly by MG132, which suppressed protein degradation, when erlotinib was added in HCC827 cells, whereasthe influence by MG132 was not evidently changed by erlotinib in H1975 cells (Fig. 2d). Furthermore, more elevation of SCD1 ubiquitination was observed in the presence of MG132 once EGFR kinase activity was repressed in HCC827but notH1975 cells (Fig. 2e). Consistently, the increase of SCD1 protein stability was observed in A549 cells after EGFR was activated (Additional file 1: Figure S2E and F). These findings together prove that EGFR stabilizes SCD1 via its kinase activity.

Since SCD1 is one of the main enzymes for MUFA synthesis, we further tested whether EGFR modulates intracellular MUFA level. The analysis by liquid chromatography-mass spectrometry (LC-MS) showed that the ratio of monounsaturated fatty acids (18:1) to saturated fatty acid (18:0) was obviously reduced by approximate 25% after the addition of erlotinib in HCC827 cells, while it remained unchangeable in H1975 cells (Fig. 2f). Likewise, the close link between EGFR activation and elevation of intracellular MUFA level was also detected in A549 cells (Additional file 1: Figure S2G). Taken together, these results demonstrate that EGFR kinase activity is critical for maintenance of SCD1 protein stability as well as intracellular MUFA level in lung cancer.

### EGFR phosphorylates SCD1 at Y14, Y41 and Y55

**Fig. 3:**
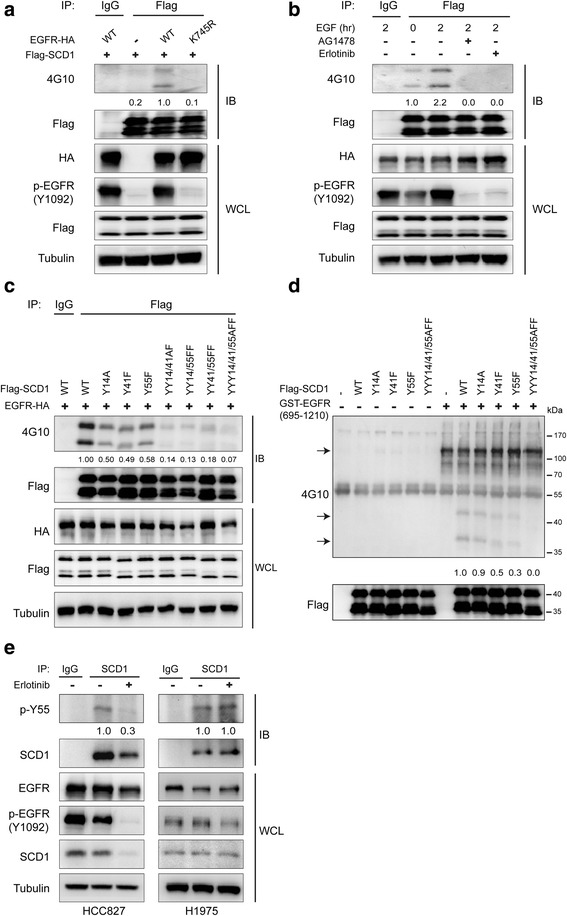
EGFR mediates phosphorylation of SCD1 at Y14, Y41 and Y55. **a** 293 T cells were co-transfected with Flag-SCD1 and wildtype or K745R mutant of EGFR-HA for 48 h. Flag-SCD1 was immunoprecipitated and its tyrosine phosphorylation was detected by a pan anti-phosphotyrosine antibody (4G10) in western blot analysis. WT, wild type. **b** 293 T cells exogenously co-expressing EGFR-HA and Flag-SCD1 were serum-starved overnight, followed by epidermal growth factor (EGF) (50 ng/ml) stimulation for 2 h with/without AG1478 (10 μM) or erlotinib (10 μM). The lysates were subjected to IP/IB with the indicated antibodies. **c** Lysates from 293 T cells ectopically co-expressing EGFR-HA and wildtype or different mutants of Flag-SCD1 were subjected to IP/IB with the indicated antibodies. **d** 293 T cells were transfected with wildtype or different mutants of Flag-SCD1 for 48 h. Flag-SCD1 was immunoprecipitated, pre-treated with lambda phosphatase and then incubated with or without a recombinant active EGFR fragment (aa 695–1210). The reaction mixtures were subjected to IB. Top arrow: EGFR fragment auto-phosphorylation; *Bottom arrows*: tyrosine phosphorylation of Flag-SCD1 and the cleaved product. **e** HCC827 and H1975 cells were treated with or without erlotinib (1 μM) for 24 h and the lysates were subjected to IP/IB with the indicated antibodies. Densitometry quantification of 4G10/Flag (**a**-**d**) and phospho-SCD1 Y55/SCD1 (**e**) levels is shown

Since EGFR can act as a tyrosine kinase to phosphorylate its binding partners, we examined whether it phosphorylates SCD1 as well. In transfected 293 T cells, we observed that wild-type but not K745R EGFR led to significant increase of SCD1 tyrosine phosphorylation (Fig. 3a). In agreement with this result, the level of SCD1 phosphorylation rose upon EGF stimulation for 2 h and dropped once EGFR kinase activity was abrogated by TKIs (Fig. 3b). It was also seen that SCD1 could be phosphorylated by various EGFR mutants as wild type (Additional file 1: Figure S3A). These data implicate that EGFR mediates tyrosine phosphorylation of SCD1.

To further identify the phosphorylation sites in SCD1, we separated SCD1 into two fragments (aa 1–216 and aa 217–359) for phosphorylation detection. Our data suggested that the N-terminal fragment, like full-length SCD1, was phosphorylated by overexpressed EGFR, while the C-terminal fragment was not (Additional file 1: Figure S3B). It needs to be noted that the C-terminal fragment was capable to bind to EGFR, which excluded the possibility that they couldn’t touch each other. On this basis, we mutated each tyrosine residue in the N-terminal fragment to check which one(s) were phosphorylated by EGFR. The representative results showed that the tyrosine phosphorylation of three mutants (Y14A, Y41F and Y55F) was reduced approximately by half when compared with wild-type SCD1 (Additional file 1: Figure S3C). It should be mentioned that mutant Y14Awas constructed in replace of Y14F, because Y14F was almost not expressed due to unknown reasons. In order to validate the result, we further generated the SCD1 constructs containing double or triple mutations of Y14, Y41 and Y55 and found that the phosphorylation of SCD1 was gradually diminished with the increasing number of mutations (Fig. 3c). In vitro kinase assay also showed that tyrosine phosphorylation of SCD1 was only observed when EGFR was added and it decreased in the mutant samples (Fig. 3d).

To detect SCD1 tyrosine phosphorylation in vivo, a specific polyclonal rabbit antibody against Y55 phosphorylation of SCD1 was generated (Additional file 1: Figure S3D). By applying this antibody, we found that the level of SCD1 Y55 phosphorylation was markedly reduced by EGFR TKIs in HCC827 cells, while it didn’t change in H1975 cells (Fig. 3e). Consistently, we detected the up-regulation of SCD1 Y55 phosphorylation by EGF stimulation in A549 cells as well (Additional file 1: Figure S3E). These evidences together prove that EGFR directly mediates tyrosine phosphorylation of SCD1 at Y14, Y41 and Y55.

### Phosphorylation of Y55 is required for maintaining SCD1 protein stability

**Fig. 4:**
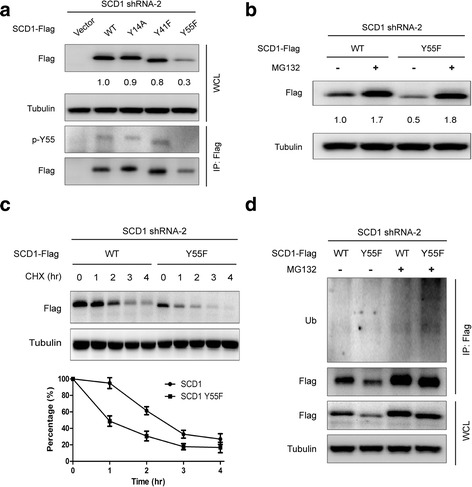
Phosphorylation of Y55 is necessary for maintainingSCD1 protein stability. **a**-**d** A549 cells were infected with SCD1 shRNA-2 lentivirus which specifically knocked down endogenously expressed SCD1 and then transfected with empty vector, ectopic wild-type SCD1 and different mutants, respectively. **a** The lysates were immunoblotted with the indicated antibodies. Y55 phosphorylation of SCD1 was examined by IP/IB. **b** A549 transfectants were incubated with/without MG132 (10 μM) for 12 h and the lysates were then subjected to IB. **c** A549 transfectants were treated with CHX (100 μg/ml) for the indicated time. The lysates were then subjected to IB. Densitometry quantitative data (Flag/Tubulin) are mean ± SEM from three independent experiments. **d** A549 transfectants were incubated with or without MG132 (10 μM) for 12 h. Then, Flag-tagged SCD1 was immunoprecipitated and blotted with an anti-ubiquitin antibody. Densitometry quantification of Flag/Tubulin (**a** and **b**) levels is shown

Having demonstrated that activated EGFR phosphorylates SCD1 at Y14, Y41, Y55 and stabilizes SCD1, we attempted to clarify whether the phosphorylation makes SCD1 more stable. For this purpose, we first stably knocked down endogenous SCD1 protein expression through the short hairpin RNAs (shRNAs) specifically targeting the UTR sequences of SCD1 in A549 cells and then re-introduced ectopic wild-type and mutated SCD1 under the same conditions. The results of western blot showed that the protein level of Y55F was lower than those of wildtype andthe other mutants (Y14A and Y41F) (Fig. 4a and Additional file 1: Figure S4A). We next tested whether Y55F mutant is deficient in maintaining SCD1 protein stability. As shown in Fig. 4b, the protein level ofSCD1 Y55F mutant was more and faster rescued by MG132 in comparison with the wildtype. In addition, SCD1 was more easily degraded if lacking Y55 phosphorylation in the presence of CHX (Fig. 4c). Furthermore, the ubiquitination of Y55F mutant was more increased than wild-type SCD1 under the treatment of MG132 (Fig. 4d).

Given that cross-talks between different post-translational modifications may bring about multiple and complicated effects, we next explored whether SCD1 phosphorylation of Y14, Y41 and Y55 functions in combination. Our results indicated that the protein stability of SCD1 YYY14/41/55AFF mutant was as similar as that of Y55F mutant, which implies that there are no combined effects of Y14, Y41 and Y55 phosphorylation on maintaining SCD1 protein stability (Additional file 1: Figure S4A and B). Additionally, we undertook similar experiments in HCC827 stable cell lines and observed coincident results (Additional file 1: Figure S4C and D). Also, it should be noted that, as shown in Fig. 4, the position of the bands representing SCD1 in gel consistently changed with the alteration of Y55 phosphorylation. Altogether, these data indicate that Y55 phosphorylation is essential for maintenance of SCD1 protein stability.

### Phosphorylation of Y55 is important for SCD1 to enhance lung cancer growth

**Fig. 5:**
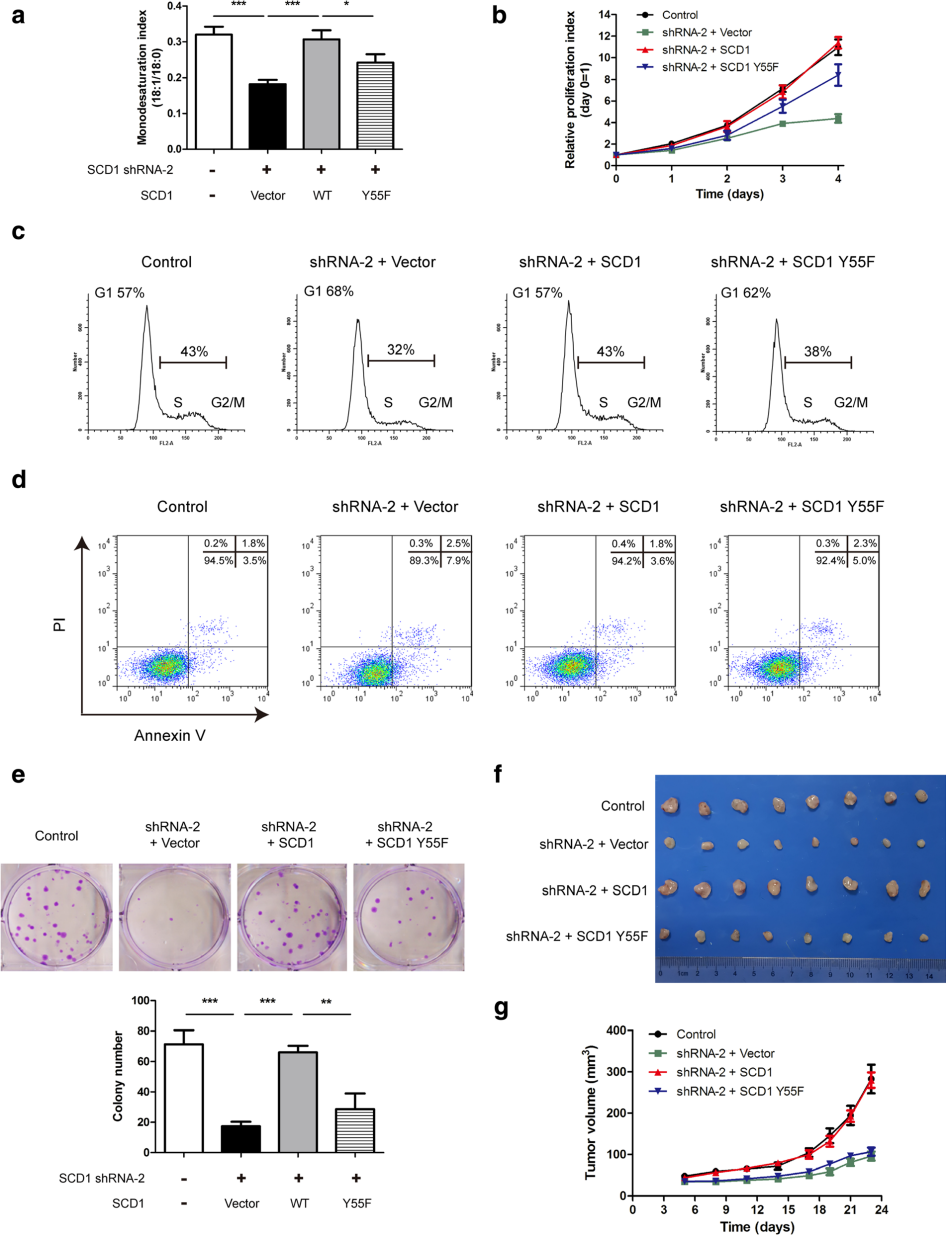
Phosphorylation of Y55 is required for SCD1 to promote lung cancer growth. **a** Total cell lipids were extracted and the ratio of monounsaturated fatty acids (18:1) to saturated fatty acid (18:0) was determined by LC-MS in A549 stable cell lines. Data are mean ± SD (*n* = 3). **b** Proliferation rates of A549 transfectants were examined by CCK-8 assay in vitro. Data are mean ± SD (*n* = 5). **c** Cell cycle of A549 stable cell lines was detected by propidium iodide (PI) staining. The percentage of cells in G1 phase or S/G2/M phase is shown. **d** Cell apoptosis of A549 transfectants was determined by Annexin V/PI staining assay. **e** In vitro growth of A549 stable cell lines was assessed by colony formation assay. The representative pictures of developed colonies are shown and the number was counted after 9 days. Data are mean ± SD (*n* = 3). **f** and **g** A549 transfectants were subcutaneously injected into nude mice and tumor volume was measured. Tumor growth curves and tumor sizes are shown. Data are mean ± SEM (*n* = 8). ***p < 0.001; **p < 0.01; *p < 0.05

Since SCD1 has been well reported to promote lung cancer growth, we sought to investigate whether Y55 phosphorylation is significant for this role. We first performed LC-MS analysis in A549 stable cell lines and found that SCD1 down-regulation evidently reduced the ratio of monounsaturated fatty acids (18:1) to saturated fatty acid (18:0) as previously reported [39, 40]. While exogenously re-expressed wild-type SCD1 could well restore this defect, Y55F mutant only partially rescued the deficiency (Fig. 5a). This implicates that SCD1 Y55 phosphorylation is necessary for de novo synthesis of MUFA in lung cancer. We also tested the metabolic profiles of these cell lines. The results showed that SCD1 did not significantly alter glycolysis (Additional file 1: Figure S5A). Consistent with previous observations, the phosphorylated AMPK, which isknown to elevate fatty acid β-oxidation, obviously increased after SCD1 was knocked down or mutated at Y55 (Additional file 1: Figure S5B). Then, we examined the cell proliferation by CCK-8 assay and observed that the cells grew slower once Y55 of SCD1 was mutated (Fig. 5b and Additional file 1: Figure S5C). In addition, our data indicated that abrogation of SCD1 Y55 phosphorylation also resulted in delay of cell cycle progression and increase of programmed cell death in lung cancer (Fig. 5c and d). Moreover, colony formation assay showed that the number of developed colonies generated from Y55F mutant re-expressing cells was lower than that from wild-type SCD1 re-expressing cells (Fig. 5e). Furthermore, in a subcutaneous mouse model, Y55F-induced tumorsgrew more slowly than the ones induced by wild-type SCD1 (Fig. 5f and g). It needs to be mentioned that triple mutations of SCD1 as YYY14/41/55AFF did not lead to more significant inhibition of cell proliferation when compared to Y55F mutation, which was in line with our above data (Additional file 1: Figure S5C). In agreement with these results, we also detected the deficiency caused by SCD1 Y55F mutation in HCC827 stable cell lines (Additional file 1: Figure S5D). Taken together, these findings demonstrate that Y55 phosphorylation is indispensable for SCD1 to facilitate lung cancer growth.

### EGFR-stimulated lung cancer growth is dependent on SCD1 activity

**Fig. 6:**
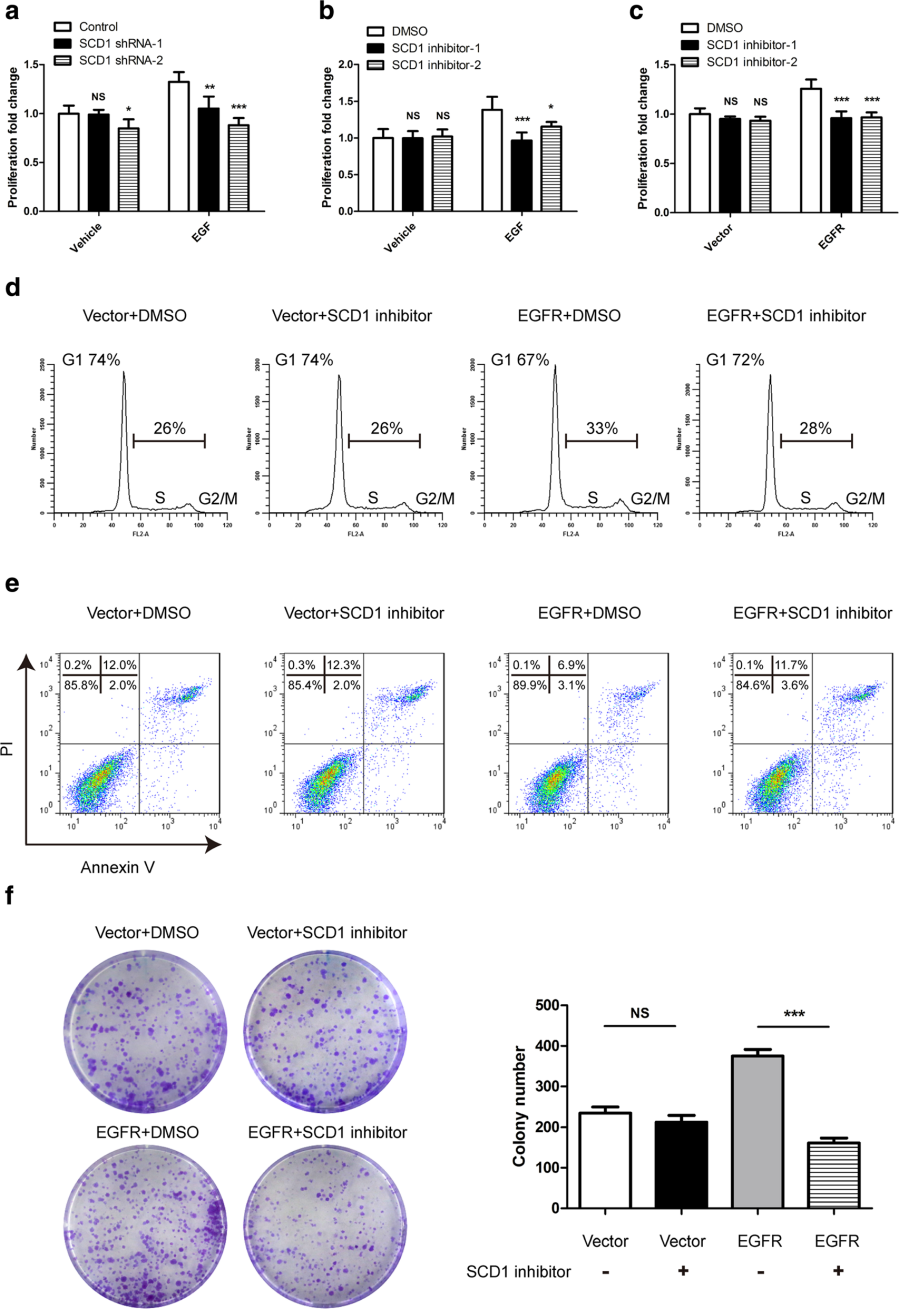
EGFR-stimulated lung cancer growth is dependent on SCD1 activity. **a** A549 stable cell lines were serum-starved overnight and then stimulated with/without EGF (10 ng/ml) for 3 days. The rates of cell proliferation were determined by CCK-8 assay in vitro. Data are mean ± SD (*n* = 5). **b** A549 cells were serum-starved overnight and then incubated with/without EGF (10 ng/ml) in the presence of 0.1% DMSO, SCD1 inhibitor-1 (MF-438) (C_19_H_18_F_3_N_5_OS) (0.1 μM) or inhibitor-2 (C_20_H_22_ClN_3_O_3_) (1 μM) for 3 days. The rates of cell proliferation were tested by CCK-8 assay in vitro. Data are mean ± SD (*n* = 5). **c**-**f** A549 stable cell lines ectopically expressing vector or EGFR were cultured in the medium containing 1% FBS, thus minimizing the influence of exogenously obtained MUFA from high concentrated FBS. **c** The transfectants were treated with 0.1% DMSO, SCD1 inhibitor-1 (0.1 μM) or inhibitor-2 (1 μM) for 2 days. The rates of cell proliferation were examined by CCK-8 assay in vitro. Data are mean ± SD (*n* = 5). **d** The transfectants were incubated with 0.1% DMSO or SCD1 inhibitor-2 (1 μM) for 24 h and cell cycle was assessed by PI staining. The percentage of cells in G1 phase or S/G2/M phase is shown. **e** The transfectants were treated as described in (**d**) and cell apoptosis was detected by Annexin V/PI staining assay. **f** In vitro growth was determined by colony formation assay. The representative pictures of developed colonies are shown and the number was counted after 11 days. Data are mean ± SD (*n* = 3). ***p < 0.001; **p < 0.01; *p < 0.05; NS, nonsignificance (when compared with the DMSO group, **a**-**c**)

Having proved that EGFR increases SCD1 protein stability through Y55 phosphorylation, which is accompanied by up-regulation of SCD1 enzyme activity, we next explored whether high-level SCD1 expression and activity are indeed important for EGFR to promote lung cancer growth. To this end, we carried out CCK-8 assay to determine the proliferation of SCD1-interfered A549 cells in the presence or absence of EGF. As shown in Fig. 6a, down-regulation of SCD1 obviously compromised EGFR-promoted lung cancer cell growth. It indicates that SCD1 serves as a critical factor in the downstream of EGFR. Interestingly, we note that SCD1 reduction had less effect on cell proliferation under the condition of serum starvation, thereby implying that SCD1 is possibly dispensable for lung cancer growth when EGFR is inactivated. Next, we used SCD1 inhibitors to block SCD1 enzyme activity in a parallel assay. Likewise, we observed that cancer cell growth was markedly suppressed by SCD1 inhibitors only in EGFR-activated cells (Fig. 6b). To further confirm the results, the growth of A549 cells stably overexpressing EGFR was compared with those infected with vector. In accordance with the above observation, the cell proliferation rate of EGFR-overexpressing cells rather than vector-overexpressing cells was evidentlydecelerated by SCD1 inhibitors (Fig. 6c). Additionally, we found that SCD1 inhibitor caused delay of cell cycle progression and enhancement of cell apoptosis in EGFR transfectants, while it had no significant effects in vector transfectants (Fig. 6d and e). Again, we observed similar results in colony formation assay (Fig. 6f). Together, these evidences prove that lung cancer growth is more dependent on SCD1 enzyme activity when EGFR is activated.

### SCD1 Y55 phosphorylation is positively correlated with EGFR activation, SCD1 protein expression and poor patient prognosis in NSCLC

**Fig. 7:**
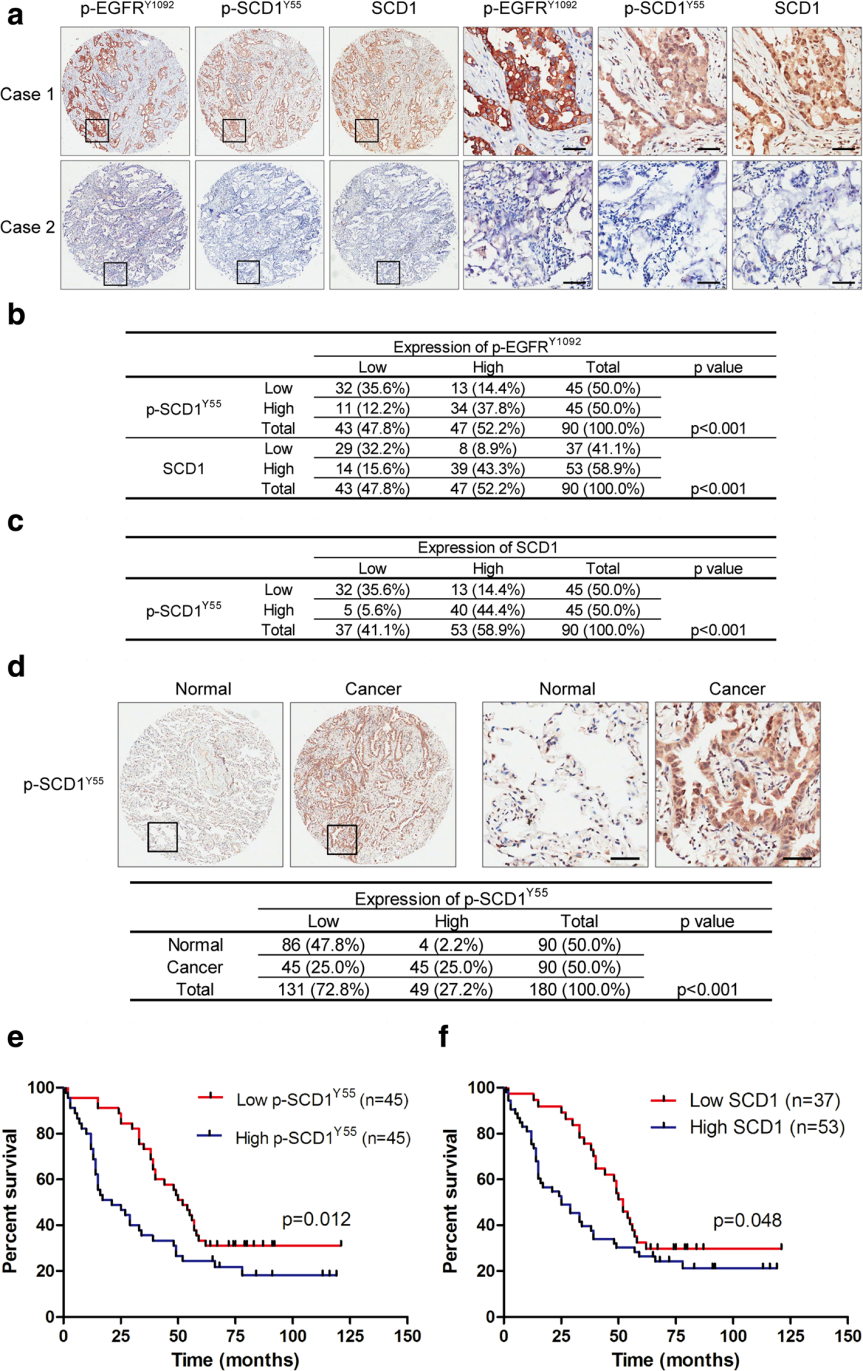
SCD1 Y55 phosphorylation is related to EGFR activation, SCD1 protein expression and poor patient survival in NSCLC. **a** Immunohistochemistry (IHC) staining of phospho-EGFR Y1092, phospho-SCD1 Y55 and SCD1 protein expression in the representative non-small cell lung cancer (NSCLC) samples with high (Case 1) or low (Case 2) levels. Scale bars represent 50 μM. **b** Correlation between phospho-EGFR Y1092 and phospho-SCD1 Y55 or SCD1 protein expression in NSCLC tissues. *p* values were calculated by chi-square test. **c** Relationship between SCD1 Y55 phosphorylation and SCD1 protein expression in NSCLC tissues. *p* values were calculated by chi-square test. **d** Representative IHC staining of phospho-SCD1 Y55 in NSCLC tissues and the paired adjacent normal tissues. *p* values were calculated by chi-square test. Scale bars represent 50 μM. **e** and **f** Kaplan-Meier analysis of overall NSCLC patient survival based on SCD1 Y55 phosphorylation or SCD1 protein expression in IHC staining. *p* values were calculated by log-rank test

To explore the clinical relationship among EGFR activation, SCD1 Y55 phosphorylation and SCD1 protein expression, we carried out immunohistochemistry (IHC) analysis of the tumor tissue microarray comprising 90 NSCLC samples. The specificity of the antibody against SCD1 Y55 phosphorylation was first verified in IHC experiment (Additional file 1: Figure S6A). The representative results showed that phospho-EGFR Y1092 was markedly associated with phospho-SCD1 Y55 and SCD1 protein expression (Fig. 7a and b). A significant correlation was also detected between SCD1 Y55 phosphorylation and protein expression (Fig. 7a and c). Moreover, the levels of both SCD1 Y55 phosphorylation and SCD1 protein expression were observed to be significantly elevated in NSCLC tissues when compared with the paired adjacent normal tissues (Fig. 7d and Additional file 1: Figure S6B). Kaplan-Meier analysis showed that high levels of phospho-SCD1 Y55, total SCD1 protein expression and phospho-EGFR Y1092 were correlated with poor prognosis of NSCLC patients (Fig. 7e, f and Additional file 1: Figure S6C). We also found that high co-expression of phospho-EGFR Y1092 and phospho-SCD1 Y55 or SCD1 was related to short survival of NSCLC patients (Additional file 1: Figure S6D and E). Furthermore, multivariate Cox regression analysis indicated that phospho-SCD1 Y55 as well as SCD1 could serve as independent prognostic factors for poor survival of NSCLC patients (Additional file 1: Figure S6F and G). These results together reveal the positive clinical relevance of EGFR activation, SCD1 Y55 phosphorylation, SCD1 protein expression and poor patient prognosis in NSCLC.

## Discussion

In this paper, we find that EGFR-mediated Y55 phosphorylation maintains SCD1 protein stability, thus increasing intracellular MUFA level to promote lung cancer growth. It is not yet clear about the details of molecular mechanism how Y55 phosphorylation interferes with the ubiquitination of SCD1. One possibility is that there may exist spatial exclusion between these two modifications in the consideration of structure, because the region of ubiquitination is possibly adjacent to Y55 site in SCD1 according to previous reports [23, 41]. Another possibility is that Y55 phosphorylation impairs the recruitment of critical factors such as E3 ligases, which are indispensable for SCD1 ubiquitination. On the other hand, though Y14 and Y41 phosphorylation don’t contribute to maintenance of SCD1 protein stability in our hands, it is still interesting to explore their roles in future studies.

As shown in our data, the growth of lung cancer cells is more sensitive to SCD1 inhibitors when EGFR is activated, which is consistent with previous finding that SCD1 activity is more essential for the cells with faster growth rate [11, 13, 15, 42]. It is also partially supported by the report that when compared with native cells, the survival of cells overexpressing EGFRvIII is more dependent on SREBP-1 activation, which is known to up-regulate SCD1 expression [43]. Thus, this evidence consolidates the importance of SCD1 in EGFR-mediatedlung cancer development and progression. Furthermore, it is promising for the application of SCD1 inhibitors in lung cancer treatment, because they may cause reduced growth suppression effects on normal cells which grow more slowly than cancer cells.

An important finding in this study is that EGFR activation, SCD1 Y55 phosphorylaion and SCD1 protein expression correlate well in the NSCLC samples, thereby supporting the clinical significance of our finding that EGFR directly modulates SCD1 protein expression through Y55 phosphorylation. We also find that the level of SCD1 Y55 phosphorylation is obviously higher in NSCLC tissues than the paired adjacent normal tissues, which validates its significant role in lung cancer. Importantly, we further reveal that in comparison with SCD1, phospho-SCD1 Y55 is a better independent prediction factor for worse prognosis of NSCLC patients due to more significant correlation. Thus, we propose that Y55 phosphorylation of SCD1 may become an ideal marker for lung cancer diagnosis.

## Conclusions

Our study uncovers a novel mechanism that EGFR directly up-regulates intracellular MUFA synthesis through phosphorylating SCD1 at Y55 to promote lung cancer growth. A positive clinical correlation among EGFR activation, SCD1 Y55 phosphorylation, SCD1 protein expression and poor patient prognosis in lung cancer further strengthens the importance of our findings. Thus, we provide the first evidence that SCD1 can be modified by tyrosine phosphorylation, thereby opening a new direction of understanding how SCD1 is controlled by other post-translational modifications. This study also reveals a previously unknown direct linkage between oncogenicreceptor tyrosine kinase and lipid metabolism in lung cancer, which is beneficial for cancer development and progression. Furthermore, we propose SCD1 Y55 phosphorylation as a potential diagnostic marker for lung cancer.

## Methods

### Cell culture

293T and A549 cells were obtained from ATCC and cultured in Dulbecco’s modified Eagle’s medium (DMEM) containing 10% fetal bovine serum (FBS). HCC827 and H1975 cells were obtained from ATCC and cultured in RPMI 1640 medium with 10% FBS. All stable cell lines were selected and cultured with puromycin (Sangon Biotech, A610593). All used cells were early passage and regularly tested to ensure free of mycoplasma contamination.

### Immunoblotting and immunoprecipitation

Cells for western blot analysis or immunoprecipitation were collected after being washed with cold phosphate-buffered saline (PBS). The pellets were lysed with mammalian protein extraction reagent (M-PER) (Thermo, 78,501) containing a cocktail of protease inhibitors (Sangon Biotech, C600387), 1 mM NaF and 1 mM Na_3_VO_4_, after discarding the supernatants by centrifugation. The lysates were then subjected to immunoblotting with the indicated antibodies. For immunoprecipitation assay, 2 mg proteins were immnoprecipitated by the specific antibodies at 4 °C overnight and protein A/G sepharose beads or anti-Flag M2 beads (Sigma, A2220) were then added for 3 h. The beads were collected and washed with lysis buffer for three times by centrifugation. Immunoprecipitated proteins were resolved by SDS-PAGE and blotted with the indicated antibodies. Software ImageJ was used for densitometry quantification of protein levels in western blot analysis.

### Fatty acid analysis

Cells were harvested in ice-cold methanol and total lipids were extracted as previously described [39]. Nonadecanoic acid (C19:0) was added as an internal standard. Pure oleic acid (18:1n-9) and stearic acid (18:0) were used as the standards. The ratio of monounsaturated fatty acids (18:1) to saturated fatty acid (18:0) was detected by LC-MS using LC20AD (Shimazhu) and 5500 QTRAP (AB SCIEX). Chromatographicpeaks were identified by comparison of the retentiontime with the standards and percent distributionwas calculated. The analysis was performed by Shanghai Applied Protein Technology Inc.

### Cell proliferation assay

Cells were seeded at the density of 2500–4000 cells/well in 96-well plates. In vitro cell proliferation was assessed by Cell Counting Kit-8 (CCK-8) (Dojindo) according to the manufacturer’s instructions.

### Cell cycle and apoptosis analysis

Cell cycle was detected by using Cell Cycle and Apoptosis Analysis Kit (Beyotime, C1052), and cell apoptosis was examined by using Annexin V-FITC Apoptosis Detection Kit (Beyotime, C1063) following the manufacturer’s instructions.

### Colony formation assay

A549 stable cell lines were plated at 350 cells per 3.5-cm dish in 10% FBS-containing medium. After 9 days, the developed colonies were stained with crystal violet and the number was counted. EGFR or vector overexpressing A549 stable cell lines were plated at 1000 cells per 3.5-cm dish and cultured in 1% FBS-containing medium. After 11 days, the developed colonies were stained with crystal violet and the number was counted.

### Xenograft model

Eight female BALB/c nude mice of 4–6 weeks old were randomly divided into each group and subcutaneously injected with 3 × 10^6^A549 stable cell lines. Tumor volume was measured by using the formula (tumor volume = ½(L × W^2^)). Tumor weight was measured after mice were sacrificed 6 weeks later.

### Tissue microarray

The microarray comprising 90 NSCLC tissues and the paired adjacent normal tissues between 2004 and 2009 were purchased from Shanghai Outdo Biotech Inc. The clinical-pathological information was provided and IHC staining was performed by the company as well.

### Statistical analysis

All in vitro experiments were repeated at least three times. Data were analyzed by Student’s t test, one-way ANOVA, Chi-square test, Kaplan-Meier survival curve analysis and Cox regression analysis. The variance was similar between the groups. A *p* value <0.05 was considered statistically significant. All statistical analyses were carried out by using software SPSS 16.0.

## Additional file

Additional file 1: EGFR modulates monounsaturated fatty acid synthesis through phosphorylation of SCD1 in lung cancer. (PDF 7425 kb)
