# Supplementary material for: EGFR modulates monounsaturated fatty acid synthesis through phosphorylation of SCD1 in lung cancer
Source: Mol Cancer. 2017 Jul 19;16:127. doi: 10.1186/s12943-017-0704-x (PMC5518108; doi:10.1186/s12943-017-0704-x)
Supplement: Additional file 1: — EGFR modulates monounsaturated fatty acid synthesis through phosphorylation of SCD1 in lung cancer. (PDF 7425 kb) [file 12943_2017_704_MOESM1_ESM.pdf]

Supplementary Figure S1

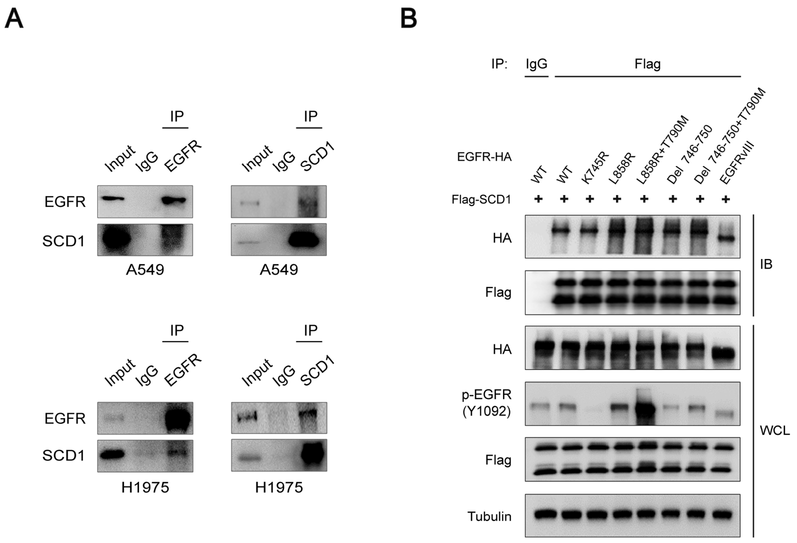

Figure S1. EGFR interacts with SCD1. (A) Reciprocal immunoprecipitation (IP) and immunoblotting (IB) by EGFR and SCD1 antibodies as indicated in A549 and H1975 cells. Ten percentage of the lysate for immunoprecipitation is shown as input. (B) Lysates from 293T cells exogenously co-expressing Flag-SCD1 and different mutants of EGFR-HA were subjected to IP/IB with the indicated antibodies.

Supplementary Figure S2

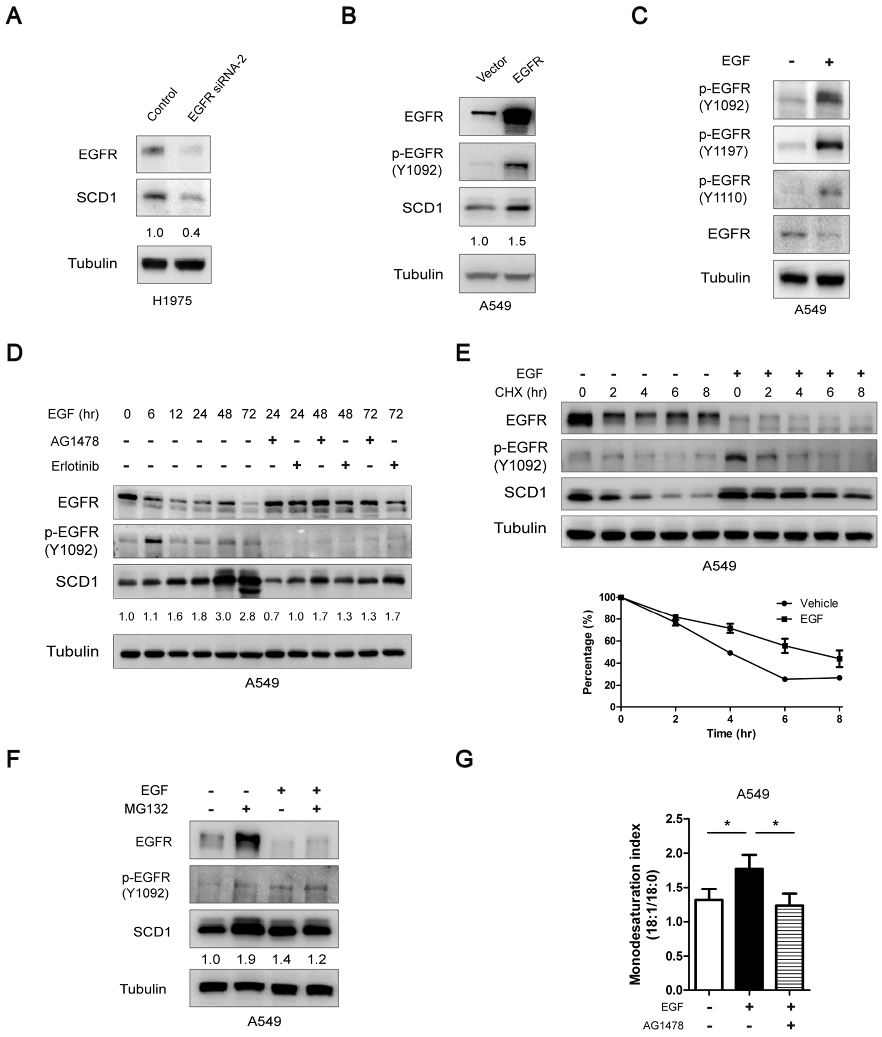

Figure S2. EGFR kinase activity is required for maintaining SCD1 protein stability and intracellular MUFA level in lung cancer. (A) H1975 cells were transfected with EGFR siRNA or a control scramble siRNA for 72 hours. The lysates were subjected to IB. (B) Lysates from A549 stable cell lines overexpressing vector or EGFR were immunoblotted with the indicated antibodies. (C) The activation of EGFR by EGF stimulation was validated by antibodies against different EGFR phosphorylation. A549 cells were serum-starved overnight and then stimulated by EGF (50 ng/ml) for 6 hours. The lysates were subjected to IB. (D) A549 cells were serum-starved overnight and then stimulated by EGF (50 ng/ml) in the presence or absence of AG1478 (10 μM) or erlotinib (10 μM) for the indicated time. The lysates were subjected to IB. (E and F) A549 cells were serum-starved overnight and then stimulated with/without EGF (50 ng/ml) for 12 hours. Next, CHX (100 μg/ml) was added for the indicated time (E) or MG132 (10 μM) was added for 12 hours (F). The lysates were subjected to IB. Densitometry quantitative data (SCD1/Tubulin) are mean ±SEM from three independent experiments. (G) A549 cells were serum-starved overnight and then stimulated with/without EGF (50 ng/ml) in the presence or absence of

AG478 (10 μM) for 24 hours. Total cell lipids were extracted and the ratio of monounsaturated fatty acids (18:1) to saturated fatty acid (18:0) was determined by LC-MS. Data are mean ±SD (n = 3). Densitometry quantification of SCD1/Tubulin protein level is shown (A, B, D and F).

Supplementary Figure S3

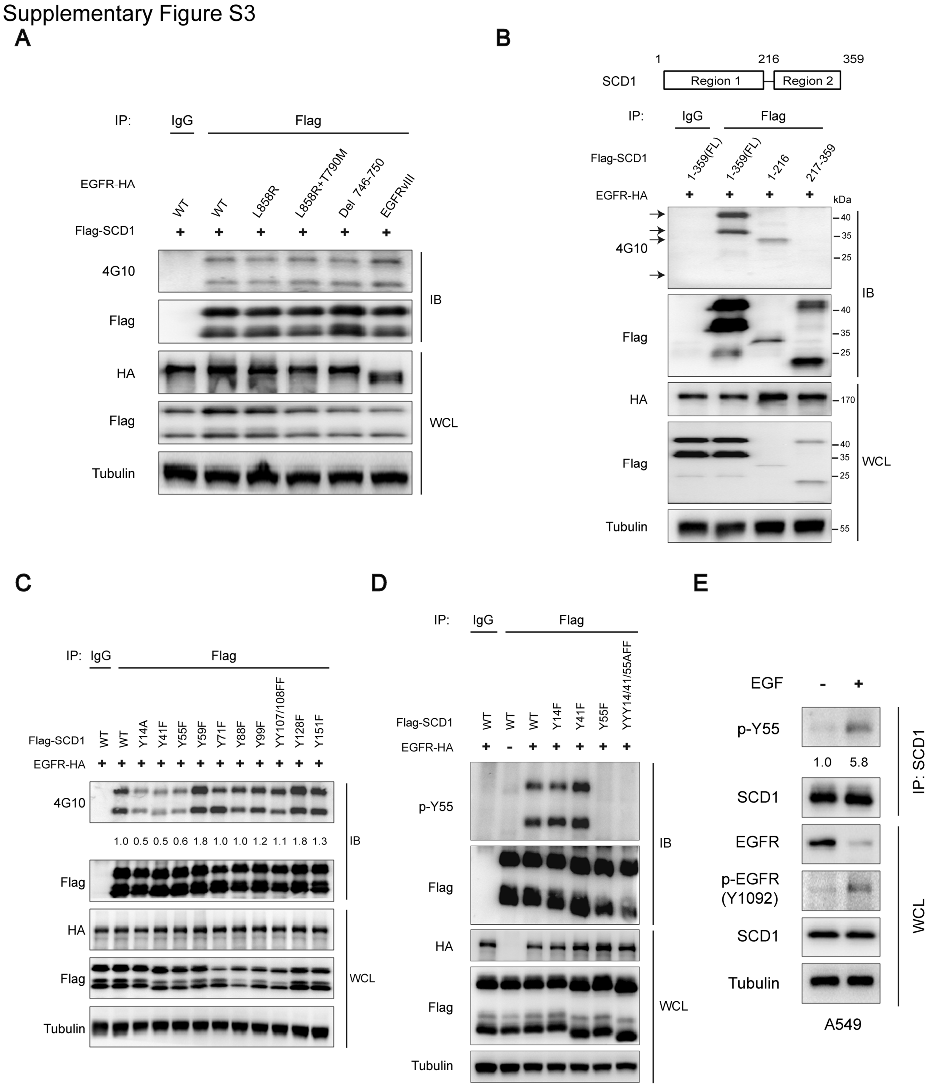

Figure S3. EGFR phosphorylates SCD1 at Y14, Y41 and Y55. (A) 293T cells were co-transfected with Flag-SCD1 and wild type or different mutants of EGFR-HA for 48 hours. The lysates were subjected to IP/IB with the indicated antibodies. (B) 293T cells were co-transfected with EGFR-HA and full-length or different regions of Flag-SCD1 for 48 hours. The lysates were subjected to IP/IB. Top two arrows: phosphorylation of wild-type Flag-SCD1 and the cleaved product; middle arrow: fragment (aa 1-216) phosphorylation; bottom arrow: fragment (aa 217-359) phosphorylation. (C) Lysates from 293T cells ectopically co-expressing EGFR-HA and wild type or different mutants of Flag-SCD1 were subjected to IP/IB with the indicated antibodies. (D) Lysates from 293T cells exogenously expressing EGFR-HA and wild type or different mutants of Flag-SCD1 were subjected to IP/IB. (E) A549 cells were serum-starved overnight and then stimulated with EGF (50 ng/ml) for 6 hours. The lysates were blotted with the antibodies as indicated. Densitometry quantification of 4G10/Flag (C) and phospho-SCD1 Y55/SCD1 (E) levels is shown.

Supplementary Figure S4

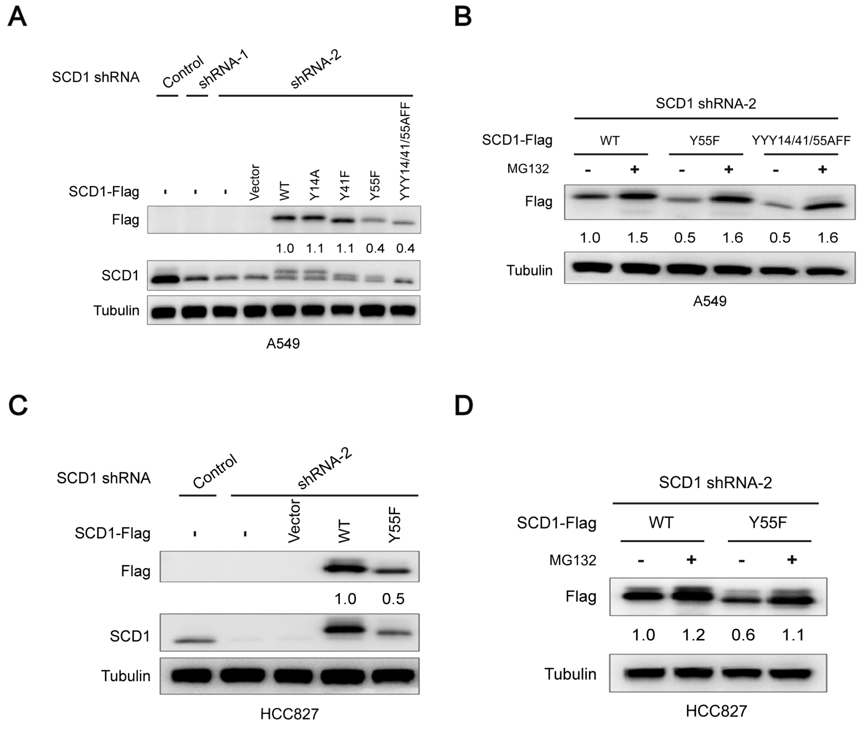

Figure S4. Phosphorylation of Y55 is essential for maintenance of SCD1 protein stability. (A) A549 cells were tranduced with shRNAs specifically knocking down endogenously expressed SCD1 and then re-introduced with empty vector, ectopic wild-type and mutated SCD1, respectively. The lysates were blotted with the antibodies as indicated. (B) A549 stable cell lines were treated with/without MG132 (10 μM) for 12 hours. The lysates were subjected to IB. (C) HCC827 cells were tranduced with SCD1 shRNA-2 which specifically knocked down endogenously expressed SCD1 and then re-introduced with empty vector, ectopic wild-type SCD1 or Y55F mutant. The lysates were blotted with the antibodies as indicated. (D) HCC827 stable cell lines were treated with/without MG132 (10 μM) for 12 hours. The lysates were subjected to IB. Densitometry quantification of Flag/Tubulin protein level is shown.

Supplementary Figure S5

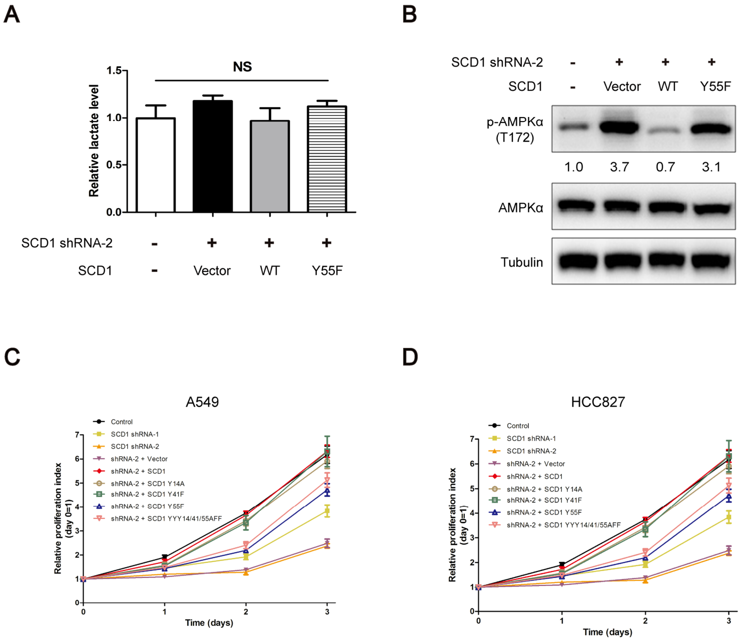

Figure S5. Phosphorylation of Y55 is necessary for SCD1 to enhance lung cancer growth. (A) The relative lactate level was determined by using a lactate colorimetric assay kit in A549 stable cell lines. The values were normalized to the cell number. Data are mean ±SD (n = 3). NS, nonsignificance. (B) The lysates of A549 stable cell lines were immunoblotted with the indicated antibodies. Densitometry quantification of phospho-AMPKαT172/AMPKα level is shown. (C and D) In vitro proliferation of A549 (C) and HCC827 (D) stable cell lines was detected by CCK-8 assay. Data are mean ±SD (n = 5).

Supplementary Figure S6

A

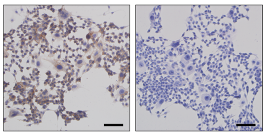

B

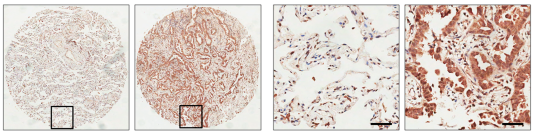

	Expression of SCD1			
	Low	High	Total	p value
Normal	85 (47.2%)	5 (2.8%)	90 (50.0%)	
Cancer	37 (20.6%)	53 (29.4%)	90 (50.0%)	
Total	122 (67.8%)	58 (32.2%)	180 (100.0%)	p<0.001

C

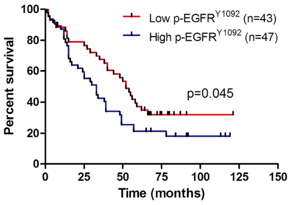

D

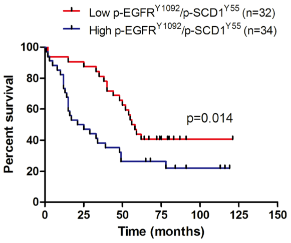

E

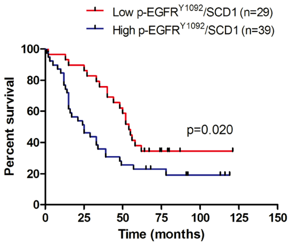

Supplementary Figure S6

F

Variable	Multivariate Analysis HR (95% CI)	p value
Gender (female vs. male)	1.2 (0.6-2.3)	0.529
Age (≤60 years vs. >60 years)	1.2 (0.7-2.2)	0.521
Tumor size (≤3cm vs. >3cm)	1.0 (0.6-1.8)	0.990
Histological grade (G1 or G2 vs. G3	0.9 (0.4-1.7)	0.702
pT status (T1 or T2 vs. T3 or T4)	1.3 (0.6-2.8)	0.456
pN status (N0 vs. N1 or N2 or N3)	4.4 (2.2-9.2)	<0.001
p-SCD1^{Y55} expression (low vs. high	3.4 (1.7-6.8)	<0.001

G

Variable	Multivariate Analysis HR (95% CI)	p value
Gender (female vs. male)	1.1 (0.6-2.0)	0.840
Age (≤60 years vs. >60 years)	1.0 (0.5-1.8)	0.986
Tumor size (≤3cm vs. >3cm)	1.1 (0.6-2.0)	0.794
Histological grade (G1 or G2 vs. G3	0.9 (0.4-1.7)	0.687
pT status (T1 or T2 vs. T3 or T4)	1.3 (0.6-2.8)	0.463
pN status (N0 vs. N1 or N2 or N3)	3.0 (1.6-5.8)	0.001
SCD1 expression (low vs. high)	1.9 (1.1-3.4)	0.033

Figure S6. SCD1 Y55 phosphorylation and SCD1 serve as independent prognostic factors for poor survival of NSCLC patients. (A) IHC staining of SCD1 Y55 phosphorylation with or without a specific blocking peptide containing phospho-SCD1 Y55 in HCC827 cells. Scale bars represent 5 μM. (B) Representative IHC staining of SCD1 in NSCLC tissues and the paired adjacent normal tissues. p values were calculated by chi-square test. Scale bars represent 50 μM. (C) Kaplan-Meier analysis of overall NSCLC patient survival based on EGFR Y1092 phosphorylation in IHC staining. p values were calculated by log-rank test. (D and E) Kaplan-Meier analysis of overall NSCLC patient survival based on co-expression of phospho-EGFR Y1092 and phospho-SCD1 Y55 or SCD1 in IHC staining. p values were calculated by log-rank test. (F and G) Multivariate analysis for overall survival of NSCLC patients. Hazard ratios (HRs), 95% confidence intervals (CIs) and p values were calculated by Cox regression analysis.

Supplementary materials and methods

Antibodies and reagents

The antibodies used were as follows: HA (Cell Signaling, 3724), Flag (Cell Signaling, 8146), α-Tubulin (Millipore CP06), SCD1 (Abcam, ab19862), EGFR (Santa Cruz, sc-03), phospho-EGFR Y1092 (Abcam ab40815), phospho-EGFR Y1197 (Santa Cruz, sc-57545), phospho-EGFR Y1110 (Santa Cruz, sc-81490), pan-anti-phosphotyrosine antibody (4G10) (Millipore, 05-321X), Ubiquitin (Abcam, ab7780), AMPKα (Cell Signaling, 5831), phospho-AMPKαT172 (Cell Signaling, 2535). The HRP conjugated secondary antibodies for western blot were obtained from Santa Cruz. The rabbit anti-phospho-SCD1 Y55 antibody was produced against a phosphorylated synthetic peptide (EDDIRPDIKDDIY(p)DPTYKDKEG) from Shanghai Ruijing Biotech Inc. The reagents used were as follows: AG1478 (Millipore, 658552), erlotinib (Santa Cruz, sc-202154), rhEGF (epidermal growth factor recombinant human) (R&D Systems, 236-EG), cycloheximide (CHX) (Sigma, C4859), MG132 (Millipore, 474790), SCD1 inhibitor-1 (MF-438) ($C_{19}H_{18}F_3N_5OS$) (Millipore, 569406), SCD1 inhibitor-2 ($C_{20}H_{22}ClN_3O_3$) (Abcam, ab142089).

Plasmid construction

For transient expression in cells, human SCD1 and EGFR cDNAs were amplified by PCR and cloned into pcDNA3.1 and pPyCAGIP vectors with different tags. For stable expression in cells, PCR-amplified human SCD1 and EGFR cDNAs were cloned into pCDH lentivirus expression vector.

Establishment of stable cell lines

293T cells were co-transfected with psPAX2 packaging plasmid, pMD2.G envelope plasmid and pCDH lentivirus expression plasmid by using Lipofectamine 2000 (Invitrogen) according to the manufacturer's instructions. The target cells were infected with lentivirus and then selected with puromycin (1 μg/ml for A549 or 0.5 μg/ml for HCC827 cells) for 2 weeks. The antibiotic-resistant cells were expanded under selective conditions for analyses.

RNA interference

All siRNAs were purchased from GenePharma Inc. Cells were transfected with 100 nM siRNAs by using Lipofectamine RNAiMax (Invitrogen). The sequences of EGFR siRNAs were as follows: 5'-CGCAAAGUGUGUAACGGAAUA-3' (siRNA-1)[1];
5'-GCAAAGUGUGUAACGGAAUAGGUAU-3' (siRNA-2)[2];
5'-CCUAUGCCUUAGCAGUCUUAUCUAA-3' (siRNA-3)[3].
The stable SCD1 knockdown cell lines were established by using lentiviral vector pGLVU6/Puro purchased from GenePharma Inc. The targeting sequences of SCD1 shRNAs were in SCD1 UTR as follows:
5'-GCAGAATCCCTTTGCACCTGA-3' (shRNA-1);
5'-GGTTCTCCAAGAAACTGAATG-3' (shRNA-2).

In vitro kinase assay

293T cells were transfected with wildtype or different mutants of Flag-SCD1 for 48 hours. Then, the cells were lysed by M-PER without phosphatase inhibitors and immunoprecipitated with an anti-Flag antibody as described in experimental procedures. The beads-bound Flag-SCD1 was washed with lysis buffer for three times by centrifugation and incubated with Lambda Protein Phosphatase (NEB, P0753) at 30℃ for 30 minutes. The dephosphorylated slurry was first washed by the buffer (20 mM Tris-HCl pH 7.5, 150 mM NaCl, 1 mM EDTA, 1% Triton X-100, 0.1% SDS, protease inhibitor cocktail, 1 mM NaF and 1 mM Na3VO4) for three times and then washed with 1 × kinase buffer (Cell Signaling, 9802) for three times. Next, it was incubated with or without 0.2 μg GST-tagged human EGFR active fragment (Sigma, SRP5023) in 50 μl kinase buffer containing 0.2 mM ATP (Sangon Biotech) at 30℃ for 30 minutes. The reaction mixtures were separated by SCD-PAGE and subjected to western blot analysis.

Lactatemeasurement

Cells were seeded in 96-well plates and the medium was replaced after enough time for cells to adhere. After culture for 24 hours, the medium was collected and the amount of lactate was determined by using a lactate colorimetric assay kit (BioVision, K607-100) according to the manufacturer's instructions. The total number of cells was counted and used to normalize lactate values. Each sample was tested in triplicate in one experiment.

Supplementary references

1 Weihua Z, Tsan R, Huang WC, Wu Q, Chiu CH, Fidler IJ *et al*. Survival of cancer cells is maintained by EGFR independent of its kinase activity. Cancer Cell 2008; **13**: 385-393.

2 Chen G, Kronenberger P, Teugels E, De Greve J. Influence of RT-qPCR primer position on EGFR interference efficacy in lung cancer cells. Biol Proced Online 2011; **13**: 1.

3 Nishimura M, Shin MS, Singhirunnusorn P, Suzuki S, Kawanishi M, Koizumi K *et al*. TAK1-mediated serine/threonine phosphorylation of epidermal growth factor receptor via p38/extracellular signal-regulated kinase: NF-{kappa}B-independent survival pathways in tumor necrosis factor alpha signaling. Mol Cell Biol 2009; **29**: 5529-5539.
